# Globular protein stabilized nanoparticles for delivery of disulfiram: fabrication, characterization, *in vitro* toxicity, and cellular uptake

**DOI:** 10.1039/c9ra09468g

**Published:** 2019-12-23

**Authors:** Muhammad Asim Farooq, Lei Li, Amna Parveen, Bo Wang

**Affiliations:** Department of Pharmaceutics, School of Pharmacy, China Pharmaceutical University Nanjing Jiangsu 211198 PR China bwangcpu@163.com +86-13913990858; The First Peoples Hospital of Xuzhou Xuzhou Jiangsu 221002 China; College of Pharmacy, Gachon University Hambakmoero, Yeonsu-gu Incheon 406-799 Republic of Korea amnaparvin@gmail.com +82-10-5925-2733

## Abstract

Disulfiram (DSF), an FDA-approved anti-alcoholic drug, has recently shown that it possesses anti-cancer effects. However, DSF is hydrophobic in nature with less stability. Therefore, new approaches are required for the effective delivery of DSF to treat cancers. Herein, we prepared DSF loaded soy protein isolate (SPI) nanosuspension (Ns) for enhancing the anti-cancer delivery of DSF. The optimized DSF-SPI-Ns had an average particle size of 164.28 ± 2.07 nm with a narrow size distribution of 0.217 ± 0.035 and zeta potential around −22.30 ± 2.11 mV, respectively. The highest drug loading and entrapment efficiency achieved was 5.516 ± 1.98%, and 91.61 ± 1.15%, respectively. The surface morphology of Ns was revealed by TEM, and the FTIR DSC, PXRD, and TGA were used for physicochemical characterization. Further, fluorescence spectroscopy and molecular docking studies were carried out to understand the interactions between (SPI and DSF) and binding sites of DSF on the surface of SPI, respectively. *In vitro* release studies showed a sustained release pattern and followed a Fickian diffusion release from the Ns. The *in vitro* cytotoxicity of SPI indicated the excellent biocompatibility, and DSF-SPI-Ns were found to be more cytotoxic compared to the free DSF solution. Moreover, the cellular uptake studies also indicated the effective delivery of the formulation to the cancer cells. Results of the current study suggested that the SPI coated Ns might be a promising drug delivery system for hydrophobic DSF, and the potential application of SPI as a coating/stabilizing agent for the delivery of hydrophobic/hydrophilic cancer therapeutics.

## Abstract 2

Disulfiram (DSF), an FDA-approved anti-alcoholic drug, has recently shown that it possesses anti-cancer effects.

## Graphical abstract

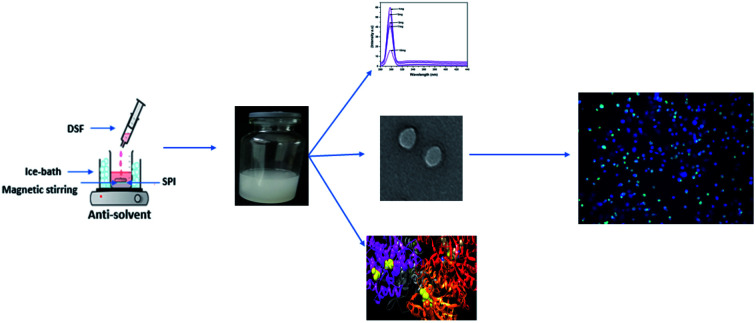

## 1. Introduction

Nanosuspensions (Ns) are colloidal dispersions of nano-sized drug particles stabilized by polymers/or surfactants. Ns-based drug delivery systems (DDs) have been considered as a promising vehicle for the efficient delivery of poorly soluble drugs.^1,2^ The methods for the formulation of NS are classified into the two categories, which are bottom-up and top-down.^3^ Currently, the top-down method has already gained more attention in business technology for Ns production.^4^ Ns formulations of various drugs are already marketed products such as Rapamune®, Megace ES®, Emend®, Tricor®, and Triglide®.^5^ Some disadvantages associated with the top-down technology are higher energy, time consumption, and less uniformity of particle size (PS).^6^ Meanwhile, bottom-up technology has many advantages, such as lower energy, easy method of preparing formulation, and smaller PS.^7,8^ Anti-solvent precipitation ultrasonication is one of the bottom-up methods widely used for preparing of Ns. Briefly, the drug is dissolved in the organic solvent, and then the solution is rapidly mixed with anti-solvent (stabilizer solution), and the supersaturated drug becomes crystal. This method is simpler and more appropriate for lab-scale investigations.^9,10^ The advantages of Ns include high encapsulation of drugs, minimal use of organic solvents, better stability, and lower toxicity in contrast to the polymeric nanoparticles (NPs), liposomes, and lipid NPs.^11^

Up to now, various drug delivery systems based on proteins, including gelatin, albumin, and whey, have been utilized for encapsulation of nutrients, food, and drugs.^12,13^ Proteins-based DDs are recognized for higher tumor penetration, better cellular uptake as compared with conventional antineoplastic agents.^14^ As compared with synthetic polymers, proteins-based Ns have less toxicity and excellent biodegradability, which make them an emerging and promising vehicle for the delivery of anti-cancer drugs.^15,16^ Among them, soy protein isolate (SPI) is one of the most promising candidates for the fabrication of nanoparticles as DDs.^17,18^

**Fig. 1:**
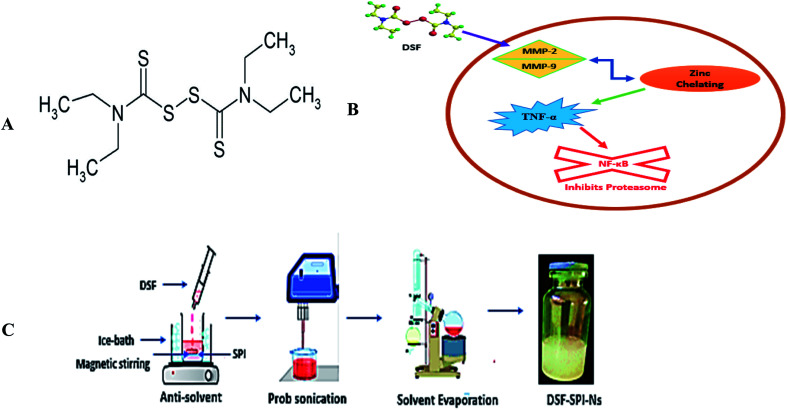
(A) Chemical structure of DSF (B) schematic illustration of the anti-cancer mechanism of DSF (C) schematic illustration of the preparation of DSF-SPI-Ns.

SPI is obtained from the soybeans and considered an excellent vehicle for drug delivery systems. There are numerous uses of SPI in the food industry due to its excellent functional properties, non-toxicity, high-nutrition values, inexpensive, natural abundance, and importantly, being considered as generally regarded as safe (GRAS) and also approved by food and drug administration (FDA) for human consumption.^19–21^ SPI is poorly water-soluble due to the presence of hydrophobic amino acids, so in this study, SPI was heated to expose the hydrophobic bonds, improve its solubility, and stabilizing effect.^20^ Disulfiram (Fig. 1A) as anti-alcoholic drug approved by the US FDA, inhibits aldehyde dehydrogenase (ALDH) enzyme.^22^ Several experimental investigations confirmed that DSF exhibited anti-cancer activity on different types of cancer, such as breast cancer, brain tumor, cervical, and prostate cancer.^23–26^

Several proposed mechanisms of DSF have been reported regarding its anti-cancer activity. The anti-cancer activity of DSF is related to the interactions with matrix metalloproteinase (MMP-2 and MMP-9) and inhibition of the proteolytic activity through a zinc chelating mechanism. DSF has shown *in vitro* cancer activity in cancer cells and inhibits the proteasome and NF-κB activity besides TNF-α-induced nuclear factor-κB (NF-κB) translocation (Fig. 1B).^27^

Up to now, there is no study reported for preparing Ns of the anti-alcoholic drug, DSF, for the repurposing of its cancer delivery to the breast cancer cells. This is the first attempt utilized for the fabrication of environmental-friendly DSF-SPI-Ns using SPI as a novel stabilizer. Ns were prepared by the anti-solvent precipitation ultrasonication method, and the optimized formulation was selected based on the optimum proportion of drug and sonication time. Fluorescence spectroscopy was carried out to investigate the interactions between DSF and SPI particles, and a docking study was also utilized to determine the binding location of DSF on the SPI. Optimized formulation was subjected to physiochemical studies by TEM, FTIR, DSC, PXRD, and TGA analysis. Further, *in vitro* drug release studies and kinetic models were employed to understand the release mechanism of the drug from Ns. Finally, the cytotoxic studies of SPI, free DSF, and DSF-SPI-Ns were investigated through MTT assay, and CLSM and FCM were used to investigate the cell uptake of Ns in breast cancer cells.

## 2. Materials and methods

### 2.1. Materials

Disulfiram (purity 99.5%) was obtained from Chemson Industrial Co., Ltd. (Shanghai, China). Soy protein isolate (SPI, CAS: 9010-10-0) was obtained from Shanghai Macklin Biochemical Co., Ltd. (Shanghai, China). Mannitol was obtained from Shandong Chuangying Chemical Co., (Shandong, China). MTT and DAPI were obtained from Sigma-Aldrich (USA). Fetal bovine serum (FBS), and Dulbecco's modified Eagle's medium (DMEM) were obtained from Thermo Fisher Scientific, Inc. (MA, USA). MDA-MB-231 breast cancer cells obtained Shanghai Cell Resource Center of the Shanghai Institute for Biological Sciences (Shanghai, China). Fluorescein isothiocyanate (FITC) was obtained from Solarbio Science & Technology Co., Ltd. (Beijing, China). The dialysis membrane (3.5 kDa) was purchased from the Shanghai Gene Ray Biotech. Co., Ltd (Shanghai, China).

### 2.2. Method

To improve the stabilizing efficiency of SPI with DSF, SPI was heated before use to expose the hydrophobic moieties buried within the SPI. Briefly, 40 mL of water and 40 mg of SPI were added into a 50 mL beaker, and then the SPI solution was stirred for 30 min at 25 °C. Next, the protein solution pH was adjusted to 7 with 0.1 M NaOH and immersed in the water bath at 105 °C for 35 min. The denatured SPI solution was cooled at room temperature for 1 h before further use.

The DSF-SPI-Ns were fabricated by an anti-solvent precipitation-ultrasonication technique (Fig. 1C). Briefly, the DSF was dissolved in 1 mL of methanol and rapidly mixed with 10 mL of SPI solution (1 mg mL^−1^) at less than 4 °C at 1200 rpm for 5 min. After the anti-solvent precipitation process, the mixture was directly prob sonicated (JY92-II, Shanghai Xinyi Biotechnology Co. Ltd., China) at 450 W. During the whole sonication process, the temperature was controlled with ice bath less than 4 °C. Finally, DSF-SPI-Ns were put under reduced pressure with a rotating speed of 45 rpm at 37 °C for 20 min to remove the residual methanol.

To trace the cellular uptake of Ns, the DSF-SPI-Ns were labeled with FITC, and the FITC-DSF-SPI-Ns were prepared as follows: 1 mL of DMSO containing 1 mg FITC was added into the 10 mL Ns and the mixture was stirred overnight at 4 °C in the dark. Then the free FITC was removed by the dialysis method and used for the *in vitro* cell uptake experiment.

### 2.3. Physicochemical characterization of SPI-DSF Ns

#### 2.3.1. Determination of particle size, polydispersity index (PDI) and zeta potential

The particle size and PDI were analyzed by dynamic laser scattering (DLS) using a Particle Size Analyzer (Brookhaven, USA) at 25 °C and a scattering angle of 90°. The mean size and PDI were calculated by using the (BIC) Dynamic Scattering Software (Brookhaven Instrument Corp., NY, USA). All measurements were determined in thrice and represented as mean ± standard deviation (SD).

#### 2.3.2. Freeze drying of Ns

To convert the DSF-SPI-Ns into dried re-dispersible powder, optimized DSF-SPI-Ns, 5 mL was added into the glass vial, followed by the addition of mannitol (10 mg) as the cryoprotectant. All the samples were pre-frozen at −20 °C for 24 h and lyophilized at −55 °C for 48 h using a freeze dryer (Shandong Laboratory Instruments Co. Ltd., China).

#### 2.3.3. Re-dispersibility index (RDI) of freeze-dried-Ns

The re-dispersibility assessment was carried out by dissolving the freeze-dried-Ns (2 mg) in (2 mL) distilled water, and resultant Ns were used for the measurement of PS and PDI. RDI was calculated by the following equation:

1 RDI (%) = *D*/*D*_0_ × 100

where *D* indicates the value of the sample after freeze-drying, and *D*_0_ indicates the value of the sample pre-freeze-drying.

#### 2.3.4. Drug loading (DL%) and encapsulation efficiency (EE%)

The DL% and EE% were harvested by ultracentrifugation (15 000 rpm) using an Optima L-100 XP Ultracentrifuge (Beckman Coulter, USA) for 15 min at 4 °C. The concentration of DSF in the supernatants was measured by UV spectrophotometer (UV-2000 spectrophotometer (Agilent Technologies Co., Ltd. USA)) at 275 nm.^28^

Finally, the DL and EE of Ns were calculated according to the following equations

2 DL (%) = DSF encapsulated/amount of SPI × 100

3 EE (%) = DSF encapsulated/amount of total drug added × 100

#### 2.3.5. Stability study of Ns

Freeze-dried optimized Ns were dispersed in the distilled water and kept at 4 °C in the refrigerator. The PS and PDI of samples were determined at predetermined times (0, 7, 14, 21, and 28 days). All the measurements were repeated in triplicate.

#### 2.3.6. Morphology analysis

The surface morphology of freshly prepared and optimized freeze-dried Ns was detected through a transmission electron microscope (TEM) (Hitachi Ltd, Tokyo, Japan) at an acceleration voltage of 100 kV. Briefly, after 15-fold dilution in purified water, one drop of Ns was dropped onto a carbon mesh and dried at 25 °C for 15 min, followed by removing an extra sample with filter paper. Next, one drop of 1% phosphotungstic acid was used to stain the sample for 5 min.

#### 2.3.7. Fluorescence spectra measurement

The fluorescence spectra were collected from the RF-5301PC Spectrofluorometer (Shimadzu, Japan) at 25 °C. The excitation wavelength was fixed at 297 nm with emission spectra recorded from 280–450 nm. The excitation and emission slits widths were set up at 5 and 10 nm, respectively. The protein concentration was 1 mg mL^−1^.

#### 2.3.8. Docking study

The docking studies were carried out using Autodock 4 (version 4.2.6) to explore the binding sites of DSF on SPI. The 3D structure of the protein and DSF (CID 3117) was obtained from Protein Data Bank in Europe and https://pubchem.ncbi.nlm.nih.gov/.

#### 2.3.9. Solid-state characterization

##### 2.3.9.1. FT-IR study

The raw DSF, SPI, mannitol, physical mixture, and optimized freeze-dried Ns were utilized for FT-IR investigation. Approximately 5 mg sample was loaded on a Burker Tensor 27 (Burker, Germany). The infrared spectrum was recorded from the wavenumber of 400 to 4000 cm^−1^ at a resolution of 4 cm^−1^.

##### 2.3.9.2. Differential scanning calorimetry (DSC) analysis

Thermal studies of raw DSF, SPI, mannitol, physical mixture (PM), and freeze-dried optimized Ns were performed using a DSC 204 (Netzch, Germany). Under a nitrogen atmosphere, 100 mL min^−1^, at a heating rate of 10 °C min^−1^ and heating range for samples 50–300 °C.

##### 2.3.9.3. Powder X-ray diffraction (PXRD)

To determine the crystalline state of DSF in Ns, DSF, SPI, mannitol, PM, and optimized freeze-dried Ns were characterized by PXRD using a D8 X-ray diffractometer (Burker, Germany) with a Cu Kα radiation detector (40 kV/40 mA) at a scan rate of 1 min over a 2*θ* range of 3.0 to 40°.

##### 2.3.9.4. Thermo-gravimetric analysis (TGA)

To determine the thermal stability of the pure drug, PM, and optimized Ns, a TGA investigation was performed on the thermogravimetric analyzer (TGA-4000, PerkinElmer, USA). The samples were heated within the range of 30–300 °C at a heating rate of 10.00 °C min^−1^.

#### 2.3.10. *In vitro* release test and kinetics analysis

The *in vitro* DSF release of DSF powder and DSF-SPI-Ns were performed with a dialysis membrane (MWCO = 3500 kDa).^29^ The phosphate buffer saline (PBS) pH 5.5 and 7.4 was used as a release medium. 2.5 mg of DSF powder or DSF-SPI-Ns were dispersed in 3 mL of distilled water and incubated in 100 mL of PBS pH 5.5 and 7.4 at 37 °C with constant stirring speed (100 rpm). Samples (3 mL) were drawn at specific intervals (0.3, 1, 2, 4, 6, 8, 10, 12, and 24 h) and replaced with the same amount of PBS heated at 37 °C. The amount of DSF was measured by UV-spectroscopy at 275 nm and the cumulative percentage release of DSF was calculated by the following equation,

4 Release (%) = DSF_released_/DSF_loaded_ × 100

Meanwhile, the release kinetics of DSF from the DSF-SPI-Ns under PBS pH 5.5 and 7.4 were further investigated by fitting the release data obtained from an experiment into five kinetic models: zero-order, first-order, Higuchi, Korsmeyer–Peppas and Hixson–Crowell.^30^

#### 2.3.11. Cell culture

MDA-MB-231 breast cancer cells obtained Shanghai Cell Resource Centre of the Shanghai Institute for Biological Sciences (Shanghai, China), were cultured in DMEM with fetal bovine serum (FBS) 10% and maintained at 37 °C in humidified incubator (Thermo Scientific, MA, USA) 5% CO_2._

#### 2.3.12. *In vitro* cytotoxicity study

The cell toxicity of SPI, free DSF, and DSF-SPI-Ns was evaluated by the MTT assay using MDA-MB-231 breast cancer cells. Briefly, cells were seeded in 96-well plates at the density of 5 × 10^4^ cells per well and incubated at 37 °C to allow for proper attachment of the cells to plate for 24 h. The culture medium was removed and substituted with fresh medium DMEM containing concentrations of SPI, DSF solution, and DSF-SPI-Ns (3.12, 6.25, 12.5, 25, 50, and 100 μg mL^−1^) and cells were incubated at 37 °C for 24 h. Next, 20 μL of MTT solution (5 mg mL^−1^ in PBS) was added to each well and further incubated at 37 °C for 4 h. Then, MTT was discarded, and 200 μL of DMSO (dimethyl sulfoxide) was added to dissolve the formazan crystals. The absorbance at 490 nm measured by a microplate reader (Multiskan FC, USA). The relative cell viability (%) was calculated by comparing the absorbance of formulation with that of untreated cells at 490 nm.

#### 2.3.13. Cellular experiments

##### 2.3.13.1. Cellular uptake study by confocal microscopy

MDA-MB-231 cells were cultured to about 85% confluency in 6-well plates containing coverslips with 3 mL of DMEM medium. After 24 h, the culture medium was replaced with FITC loaded Ns at a fixed concentration of 20 μg mL^−1^ and incubated at 37 °C for 1 h and 4 h. Afterward, the culture media was discarded, and the cells were washed thrice with ice-cold PBS to remove the free FITC-DSF-SPI-Ns. Next, cells were fixed with 4% paraformaldehyde (PFA) in PBS (10 min), and the nucleus of cells was stained with DAPI (5 μg mL^−1^) for 15 min at 25 °C and visualized by using confocal microscope, (Carl Zeiss, Germany).

##### 2.3.13.2. Flow cytometry (FCM)

MDA-MB-231 cells were similarly seeded in 6-well plates and co-incubated with FITC-DSF-SPI-Ns at the fixed concentration of FITC (10 μg mL^−1^). After incubation with FITC-DSF-SPI-Ns at different times (2, 4, 8, 24, and 48 h), the medium was removed, and the cells were washed with ice-cold PBS thrice to remove unbound Ns, and 0.25% trypsin was added for the digestion of cells. Finally, the cells were collected by centrifugation at 1000 rpm for 5 min and resuspended in PBS. The fluorescence intensity was measured by flow cytometer (FACS Calibur, BD Biosciences, USA).

#### 2.3.14. Statistical analysis

All the experimental tests were performed in triplicate, and the results were expressed as mean ± standard deviation (SD). Mean values were compared using analysis of variance (ANOVA), and the difference was considered statistically significant at ***P < 0.001.

## 3. Result & discussion

### 3.1. Preparation and optimization of DSF-SPI-Ns

**Fig. 2:**
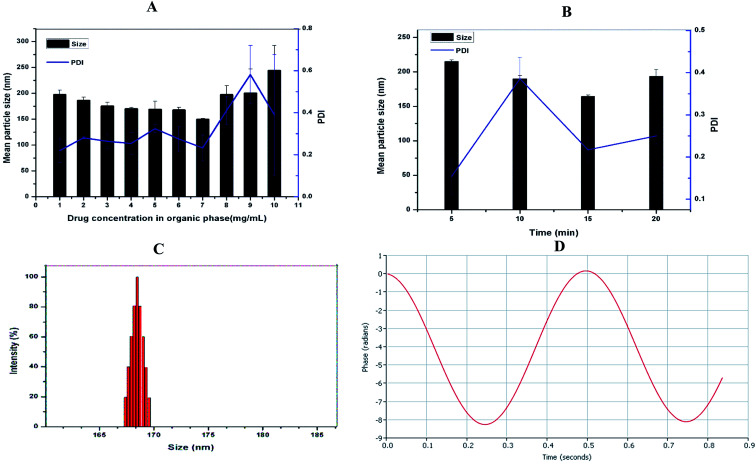
(A) Formulation optimization of DSF-SPI Ns, the influence of drug concentration in methanol, and (B) effect of the time length of ultrasonication on particle size and PDI (*n* = 3). (C) Size distribution and (D) zeta potential graph of optimized DSF-SPI-Ns.

Although native soy protein isolate is poorly soluble in water, its solubility is markedly improved by heat-induced denaturation. Also, the heat procedure reveals the hydrophobic residues suppressed within the SPI.^31^ DSF-SPI Ns were prepared by the anti-solvent precipitation–ultrasonication method, as reported previously.^32^ To obtain the optimized formulation, the impacts of drug concentration and the sonication time on particle size and PDI were studied in the current experiment. As depicted in Fig. 2A, the mean particle size, and PDI decreased as the amount of DSF in 1 mL of ethanol increased from 1 to 7 mg mL^−1^. However, further increase the drug concentration from 8 to 10 mg mL^−1^ markedly increased the particle size and PDI, respectively. The SPI coating on the drug nano-scaled particles prevented the aggregation of DSF particles, and a further increase in the drug concentration led to inadequate stabilizing effect by stabilizer; thus, larger particle size.^33^

Next, we investigated the impact of ultrasonication time on particle size and PDI, as shown in Fig. 2B. The particle size and PDI decreased as sonication time increased from 5 min to 15 min, and a further increase in sonication time could not reduce the particle size and PDI, respectively. It is already reported that higher sonication time increases the kinetic energy between nanoparticles and provides sufficient time for SPI to bind with drug particles; thus, suppressing the nanoparticle aggregation.^34^

The conditions for the optimized formulation were as follows: SPI concentration, 1% (w/v) 10 mL; the amount of DSF, 7 mg in 1 mL ethanol; power; 450 W, and sonication time 15 min.

### 3.2. Measurement of particle size, PDI and zeta potential

The particle size and charge on the surface of nanoparticles had a significant role in cell uptake and *in vitro* drug releases.^35,36^ The optimized DSF-SPI-Ns had a mean particle size 164.28 ± 2.07 nm and PDI 0.217 ± 0.035, respectively. As shown in Fig. 2C, the optimized formulation showed a narrow size distribution. As stated previously, PDI between 0.1 to 0.3 is usually considered a narrow size distribution of NPs.^37^ The zeta potential is an important parameter that can affect the stability of the developed formulation, and Stability is essential to prevent the aggregation of the NPS.^38^ In the present study, the zeta potential was −22.30 ± 2.11 mV that is sufficient for stabilizing the NPs for long term storage (Fig. 2D).

### 3.3. Effect of freeze-drying and reconstitution/RDI

**Fig. 3:**
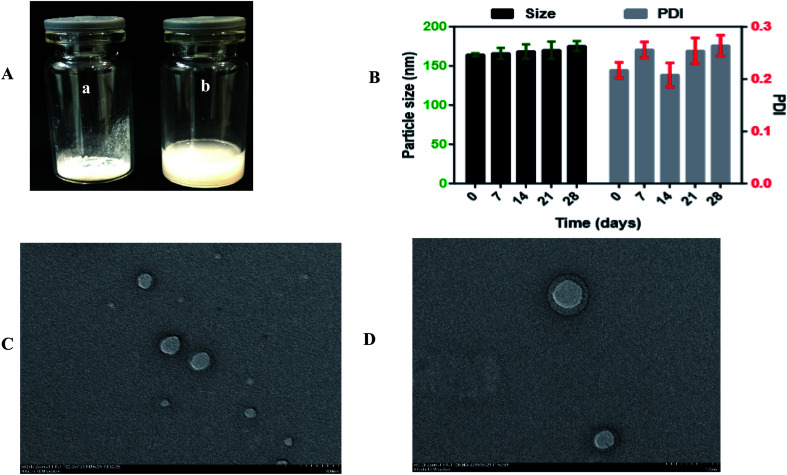
(A) Optical images of (a) freeze-dried DSF-SPI Ns, and (b) reconstituted DSF-SPI-Ns. (B) Short term stability study for 28 days at 4 °C. (*n* = 3). (C) TEM images of fresh prepared optimized DSF-SPI-Ns and (D) freeze-dried optimized DSF-SPI-Ns at scale bar 100 nm.

**Fig. 4:**
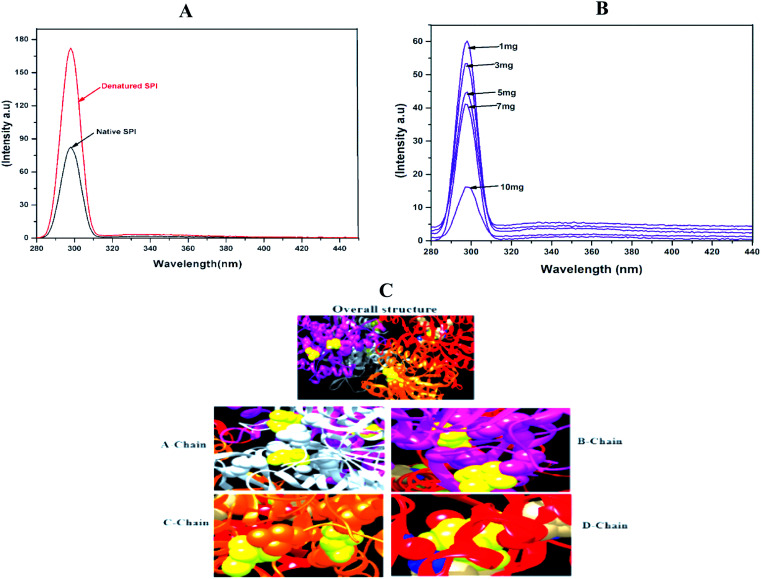
Fluorescence emission spectra of (A) native and denatured SPI and (B) different drug concentrations of DSF. Amount of SPI (1 mg mL^−1^). (C) The overall structure, and binding chains (A–D).

To explore the influence of the freeze-drying process on the optimized formulation, 1% mannitol was added as a cryoprotectant to reduce the lyophilization stress during the process. The particle size of the optimized formulation was 164.28 ± 2.07 nm and slightly increased to 167.63 ± 3.27 nm, and the PDI of the optimized formulation was 0.217 ± 0.035 and increased to 0.275 ± 0.0059. These results indicated that the particle size was stable even after the freeze-drying process, and Ns can be stored for a long-term period. Moreover, the optical images of freeze-dried Ns and reconstituted Ns are shown in Fig. 4. It is seen in Fig. 3A(a and b); there was no aggregation of particles in freeze-dried Ns and completely dispersed in distilled water upon reconstitution.

In this study, the RDI of optimized formulation was 102.03 ± 0.10%. Generally, the RDI value closer to 100% is considered for the homogeneous dispersion of NPs.^39^

### 3.4. Measurement of DL% and EE%

The drug lading and EE% of the optimized formation were determined by the spectrophotometer. The EE% and DL% achieved in this study were 91.61 ± 1.15% and 5.516 ± 198%, respectively. The high EE% and DL% might be due to the hydrophobic property of SPI and SPI-DSF interactions.^40^

### 3.5. Short term physical stability study

The stability of the optimized DSF-Ns was evaluated at 4 °C for a month. As revealed in Fig. 3B. It was found that the particle size of DSF-SPI-Ns slightly increased from 164.28 ± 2.07 nm to 175.26 ± 4.426 nm after 28 days. Besides, the PDI also changed from 0.217 ± 0.035 to 0.264 ± 0.020 with the variations of PS. The results indicated the excellent storage stability of DSF-Ns over time.

### 3.6. TEM analysis

The surface morphology of freshly prepared DSF-Ns and freeze-dried Ns is shown in Fig. 3C and D; TEM micrographs showed spherical shape, nanosized, and smooth surface without any aggregation. The smooth surface of nanoparticles also depicted the complete encapsulation of the drug during the preparation step.

The size of NPs determined by TEM is smaller than the size measured by DLS. This difference might be due to the hydrodynamic diameter of NPs based on intensity in the Ns. In contrast, TEM analysis is based on the number and also dried state of the sample during measurement. Therefore, the size of NPs resulting from TEM is always smaller than that from the DLS.^41^

### 3.7. Fluorescence spectra measurement

Fluorescence is one of the most suitable techniques for understanding the interactions between drug particles and proteins. As shown in Fig. 4A, the heat-induced denaturation increased the fluorescence intensity of SPI as compared with native SPI. The heat denaturation of SPI typically reveals the hydrophobicity of residues to a less hydrophobic microenvironment, therefore resulting in fluorescence quenching after denaturation.^42^ Besides, native SPI was poorly soluble in water, so displayed low fluorescence intensity as compared to the denatured SPI.

Meanwhile, denatured SPI had better solubility in water than native SPI and showed higher fluorescence intensity. Next, to interpret the interactions between DSF with SPI, the fluorescence emission spectra at different DSF concentrations are shown in Fig. 4B. The Trp fluorescence intensity of SPI decreased as the amount of DSF increased from 1 to 10 mg, and simultaneously, *λ*_max_ shifted to the shorter wavelength. Therefore, the SPI fluorescence quenching induced by DSF was investigated in this work.

### 3.8. Docking study

Molecular docking study was used to explore the binding affinity and the preferred location of DSF when it is bound to SPI.^43^ In this study, DSF was docked to the SPI, as presented in Fig. 4C. The SPI has a 3D globular structure with 4 chains connected with a bond length of 2.1–3.02 Å. The DSF (yellow ball) binds with A-chain at positions 412 and 1778; on the B-chain, the drug binds in the cavity exposed by 4014 and 4315, further, chain-C & D and the DSF bind at 6112, 6372 and 9255, respectively. This result confirmed the strong binding affinity between SPI and DSF.

### 3.9. FT-IR

**Fig. 5:**
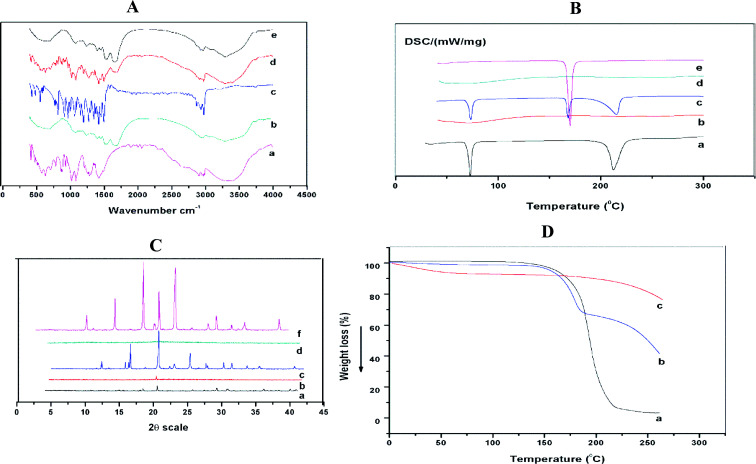
(A) FTIR spectra of (a) mannitol, (b) SPI, (c) DSF (d) physical mixture and (e) optimized formulation. (B) DSC thermograms of (a) DSF, (b) optimized formulation, (c) physical mixture, (d) SPI and (e) mannitol. (C) X-ray diffractograms of (a) DSF, (b) optimized formulation, (c) physical mixture (d) SPI and (e) mannitol. (D) TGA thermograms of (a) DSF, (b) physical mixture, and (c) optimized formulation.

FTIR spectroscopic analysis was performed to study the possible molecular interactions between disulfiram and stabilizer in the Ns. FTIR spectra of pure drug, SPI, mannitol, physical mixture (Pure DSF, SPI, and mannitol) and optimized formulation are shown in Fig. 5A. For the spectra of disulfiram (Fig. 5A(c)), the bands at 2974.1 cm^−1^ and 1496.2 cm^−1^ were ascribed to C–H–CH_3_ stretching and corresponding to C–H symmetrical deformation vibrations, respectively. The absorption peaks at 1349.1–1455.1 cm^−1^ were ascribed to CH_2_ and CH_3_ deformation.^44^ The vibrational bands at 1272.4 cm^−1^ and 1149.3–1194.5 cm^−1^ were corresponding to the C=S stretching and C–C skeletal vibrations, respectively.^45^

The spectrum of SPI showed that the most characteristic peaks appeared at 1631–1689, 1513–1546, and 1237 cm^−1^ due to C–O stretching, N–H bending, and C–H and N–H stretching, respectively.^46^ The bands at 2874.4–3080.2 cm^−1^ were ascribed to the O–H and N–H bending vibrations (Fig. 5A(b)).^47^ The IR spectra of mannitol displayed (Fig. 5A(a)) bands between 3397.1 ad 2902.7 cm^−1^ due to O–H and CH stretching vibrations, also other characteristic bands at 1419.8, 1209.3, 1080.9 cm^−1^.^48^ On the other hand, the spectrum of the physical mixture showed fused peaks of DSF without shifting the position, while in the optimized formulation, the characteristic peaks of the drug were detected, which exhibited an absence of chemical interactions.

### 3.10. Differential scanning calorimetry (DSC)

DSC thermograms of raw DSF, SPI, mannitol, physical mixture (pure DSF, SPI, and mannitol) are shown in Fig. 5B. Pure DSF had an intense endothermic peak at 70.0 °C, which indicates the melting point of pure DSF and its crystalline nature (Fig. 5B(a)).^49^ There was no sharp peak detected for SPI, which shows the amorphous state of stabilizer and mannitol had a characteristic peak at 168 °C, respectively.^50^

Furthermore, DSC thermograms of physical mixture revealed that DSF present in the crystalline state, and there was no change in the endothermic peak of mannitol. However, no such peak was observed for the optimized formulation indicating disulfiram amorphous state in the Ns.

### 3.11. Powder X-ray diffraction (PXRD)

The PXRD patterns of the raw DSF, SPI, mannitol, physical mixture (raw DSF, SPI, and mannitol) and freeze-dried DSF-Ns were performed to understand the crystalline state of the drug when in Ns. The coarse DSF powder (Fig. 5C(a)) showed diffraction peaks at 2*θ* of 14.43°, 17.350°, 19.442°, 28.12°, 28.77°, 35.793°, and 39.176°, which confirmed the crystalline state of disulfiram. For SPI, there were no intense peaks observed, which indicated the amorphous state of SPI. The peaks were showed for mannitol at 14.581°, 18.731°, 23.387°, 28.239°, 31.311°, 33.876°, and 38.640°. Furthermore, in the physical mixture, the peaks of DSF and mannitol (DSF peaks overlapping with the mannitol peaks) indicated that they contain crystalline DSF.

However, none of these peaks are observed in the optimized formulation suggesting the complete encapsulation of drug in the Ns.

### 3.12. TGA analysis

To assess the thermal behavior of raw DSF, physical mixture, and freeze-dried optimized Ns, TGA analysis was performed. Fig. 5D(a), the typical curve of free DSF showed weight loss from 190 °C and continued up to 300 °C, however, the freeze-dried optimized formulation showed minimal weight loss before 190 °C, and weight loss started at 230 °C. The weight loss of free DSF may be due to the faster decomposition rate as temperature raised. The results displayed that the physical stability of freeze-dried optimized formulation was higher than the free DSF.

### 3.13. Release of DSF from DSF-SPI-Ns and kinetics analysis

**Fig. 6:**
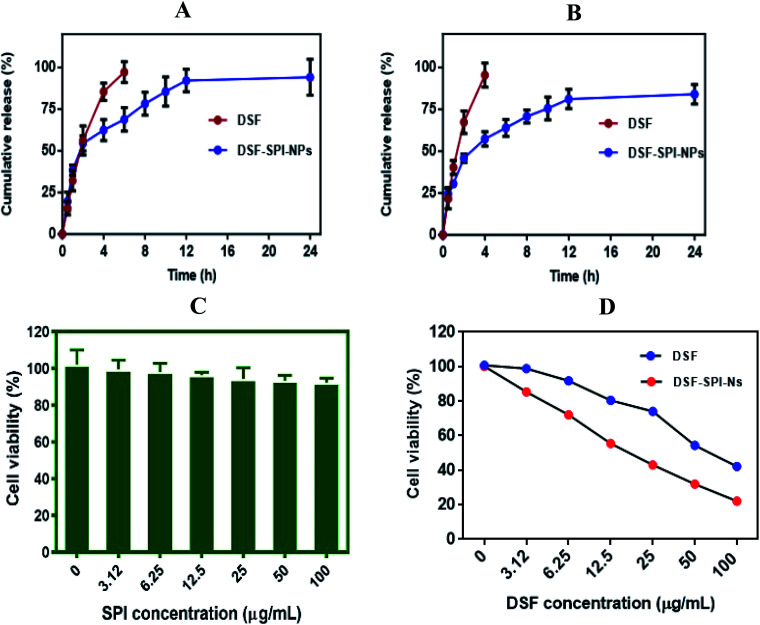
*In vitro* drug release profile of DSF from DSF-SPI-NS in PBS solutions (A) pH 5.5 (B) pH 7.4 at 37 °C for 24 h. (*n* = 3). Cell viability after incubation with (C) SPI (D) free DSF and DSF-SPI-Ns in MDA-MB-231 cells for 24 h (*n* = 3). ***P < 0.001 compared with DSF.

To compare the release profile of free DSF solution and DSF loaded SPI-Ns, we have performed *in vitro* release study using a dialysis bag, and the results were compared with the free DSF solution (Fig. 6A and B). The *in vitro* drug release was studied under PBS pH 5.5 and pH 7.4 at 37 °C for 24 h. The results of the drug release experiment demonstrated the fast diffusion of DSF from free drug solution where more than 80% and 90% drug was released within 4 h at pH 5.5 and pH 7.4, respectively. In contrast, the DSF-SPI-Ns exhibited a biphasic release trend with initial burst release followed by a sustained release pattern. In the first 2 h, over 50% and 40% drug was released from the SPI DSF-Ns at both pH values. The burst release was due to the swelling and breaking of the SPI matrix.^51^ Also, weakly adsorbed drug on the surface of the NPs might have moved to the release media during the first 2 h.^52^ Between 2 and 24 h, a sustained release behavior was observed in which the concentration of released DSF increased steadily with time. At the end of 24 h, the total DSF released from the SPI-DSF-Ns was 94.12% and 84% at pH 5.5 and 7.4, respectively. These results attributed to the diffusion of DSF within the hydrophobic core of the SPI molecules.^53^

The *in vitro* release kinetics of DSF from DSF-SPI-Ns was studied using different kinetic models. The values of the regression coefficient (*R*^2^) are shown in Table 1. The results showed that the Higuchi and Korsmeyer–Peppas models are the best fit model to explain the release pattern as the value of *R*^2^ is 0.9635 and 0.9641, respectively. The results showed that the DSF release from the DSF-SPI-Ns explains the diffusion-controlled released and Fickian diffusion mechanism.^54,55^

**Table 1:** Modeled release kinetic equations of DSF-SPI-Ns

Formulation	Zero-order	First-order	Higuchi	Korsmeyer–Peppas	Hixson–Crowell
DSF-SPI-Ns pH 7.4	*y* = 2.9985*x* + 33.135	*y* = −0.0327*x* + 1.8183	*y* = 17.783*x* + 14.386	*y* = 0.3442*x* + 1.5208	*y* = −0.0838*x* + 4.038
	*R* ^2^ = 0.6342	*R* ^2^ = 0.8121	*R* ^2^ = 0.8925	*R* ^2^ = 0.9641	*R* ^2^ = 0.7566
DSF-SPI-Ns pH 5.5	*y* = 3.4142*x* + 36.351	*y* = −0.0528*x* + 1.8141	*y* = 11.039*x* − 10.729	*y* = 0.3861*x* + 1.5326	*y* = −0.1165*x* + 3.9851
	*R* ^2^ = 0.6292	*R* ^2^ = 0.8691	*R* ^2^ = 0.9635	*R* ^2^ = 0.9089	*R* ^2^ = 0.8017

### 3.14. *In vitro* cytotoxicity of DSF-SPI-Ns

Cytotoxicity of denatured SPI, free DSF, and DSF-SPI-Ns against MDA-MB-231 cells was determined by MTT assay, and the results are shown in Fig. 6C. Cells were incubated with denatured SPI at different concentrations at 37 °C for 24 h. It is indicating that the SPI does not show any cytotoxicity against MDA-MB-231 and cell viability was higher than 90%. This higher cell viability might be attributed to the hydrophilic nature and excellent biocompatibility of denatured SPI.

In contrast, the cytotoxicity of free DSF and DSF-SPI-Ns was dose-dependent after 24 h incubation, and the cell viability was decreased with the increase of DSF concentration (Fig. 6D). However, free DSF exhibited less cytotoxic effect in cancer cells after 24 h incubation due to its low solubility and stability. This lower cytotoxic effect might be due to the rapid degradation of free DSF in culture media, and the higher cytotoxic effect of DSF-SPI-Ns might be due to its better stability and protection against reactive molecules in cell medium.^56^ In conclusion, the DSF-SPI-Ns at all the concentrations showed a superior anti-cancer effect compared with the free DSF.

### 3.15. Cellular uptake study

#### 3.15.1. Confocal microscopy study

The qualitative cellular uptake of DSF-SPI-Ns was examined using MDA-MB-231 breast cancer cells treated with free FITC solution (control), and FITC loaded DSF-SPI-Ns. The confocal microscopy was utilized to visualize the cellular uptake of FITC loaded DSF-SPI-Ns.

**Fig. 7:**
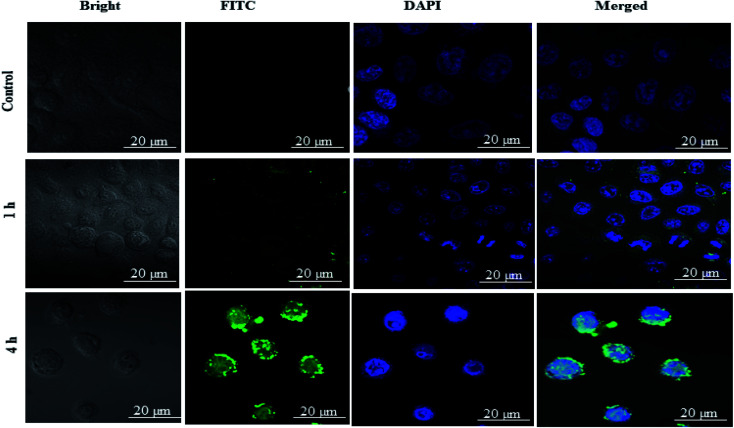
Cellular uptake of DSF-SPI-Ns. Confocal microscopy images of MDA-MB-231 cells treated with FITC-DSF-SPINs (green) for different durations at fixed FITC concentration of 20 μg mL^−1^ FITC. The nuclei (blue) were stained with DAPI. Free FITC was used as control. Scale bar: 20 μm.

As shown in the Fig. 7 free-FITC solution could not be taken up by the cancer cells and showed no fluorescence intensity (green color). In contrast, FITC loaded DSF-SPI-Ns showed an increase in cell fluorescence intensity at 37 °C for various time points (1 and 4 h) incubation. As revealed in Fig. 7, the NPs were gradually taken up by MDA-MB-231 cells in a time-dependent manner and showed stronger fluorescence over time. Moreover, as time increased to 4 h, the green fluorescence occupied the cytoplasm around the nuclei, further indicating that the NPs were gradually endocytosed into the cancer cells. In short, better cell uptake is a critical factor in evaluating drug delivery efficiency.^57^ The physicochemical properties, such as NP size and charge on the surface, played a significant role in the uptake of NPs.^58^ In this study, the NP size was less than 200 nm and more efficiently penetrated the cells, while the NPs with larger size required more driving forces and energy in the cell internalization. Most importantly, the high cellular uptake of negatively charged DSF-SPI-Ns may be due to certain peptides from SPI protein that activated the cell uptake mechanism in MDA-MB-231.^58^

#### 3.15.2. Flow cytometry analysis

**Fig. 8:**
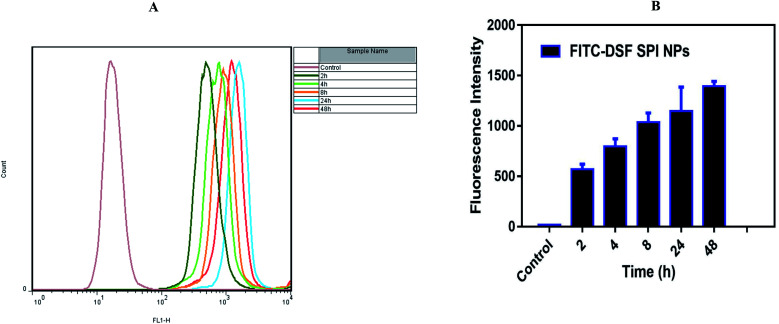
Flow cytometry images of (A and B) MDA-MB-231 cells treated with FITC-DSF-SPI-Ns for different durations at fixed FITC concentration of 20 μg mL^−1^ FITC.

The flow cytometry was employed for the quantitative cellular uptake of the FITC-SPI-DSF-Ns inside the MDA-MB-231 cells. The Ns were incubated with breast cancer cells for a different period. As shown in Fig. 8A and B, the mean fluorescence intensity increased as the duration increased, which further indicates the time-dependent trend of FITC-DSF-SPI-Ns. These results were consistent with the confocal microscopy observations.

## 4. Conclusions

In summary, we have successfully prepared DSF-SPI-Ns by the anti-solvent precipitation ultrasonication method for cancer treatment, and SPI was utilized as the stabilizing agent. Also, the nano-sized (164 nm) and sphere-shaped DSF-SPI-Ns exhibited excellent drug loading and entrapment efficiency, had better stability, and sustained drug release. Further, the drug release kinetics suggested Korsmeyer–Peppas models at physiological pH 7.4. Also, the better cytotoxic effect of DSF-SPI-Ns was observed in the MDA-MB-231 cells as compared to the free DSF drug. The *in vitro* cellular uptake investigation showed that the DSF-SPI-Ns could be efficiently internalized by breast cancer cells.

Generally, proteins are considered less toxic, biodegradable, and biocompatible, and also from this study, it is concluded that the SPI can be used for the preparation of Ns based novel drug delivery systems for various chemotherapeutic agents. However, additional investigations regarding the *in vivo* experiment should be done to further prove the suitability of these Ns.

## Funding

This research was supported by Basic Science Research Program through the National Research Foundation of Korea (NRF) funded by the Ministry of Education (NRF-2019R1G1A1003693).

## Abbreviations

ALDH: Aldehyde dehydrogenase
DLS: Dynamic laser scattering
DDs: Drug delivery systems
DMEM: Dulbecco's modified Eagle's medium
DSF: Disulfiram
DAPI: 4,6-Diamidino-2-phenylindole
FBS: Fetal bovine serum
FITC: Fluorescein isothiocyanate
FT-IR: Fourier transform infrared
MTT: 3-(4,5-Dimethylthiazol-2yl)-2,5-diphenyltetrazolium bromide
Ns: Nanosuspensions
NPs: Nanoparticles
PS: Particle size
PDI: Polydispersity index
PBS: Phosphate buffer saline
RDI: Re-dispersibility index
SPI: Soy protein isolate
TEM: Transmission electron microscope

## Conflicts of interest

There are no conflicts of interest reported by authors.

## Supplementary Material
